# Cross-species hybridisation of human and bovine orthologous genes on high density cDNA microarrays

**DOI:** 10.1186/1471-2164-5-83

**Published:** 2004-10-28

**Authors:** James Adjaye, Ralf Herwig, Doris Herrmann, Wasco Wruck, Alia BenKahla, Thore C Brink, Monika Nowak, Joseph W Carnwath, Claus Hultschig, Heiner Niemann, Hans Lehrach

**Affiliations:** 1 Max Planck Institute for Molecular Genetics, (Department of Vertebrate Genomics), Ihnestrasse 73, D-14195, Berlin, Germany; 2 Institute for Animal Science, (Department of Biotechnology), Mariensee, 31535 Neustadt, Germany

## Abstract

**Background:**

Cross-species gene-expression comparison is a powerful tool for the discovery of evolutionarily conserved mechanisms and pathways of expression control. The usefulness of cDNA microarrays in this context is that broad areas of homology are compared and hybridization probes are sufficiently large that small inter-species differences in nucleotide sequence would not affect the analytical results. This comparative genomics approach would allow a common set of genes within a specific developmental, metabolic, or disease-related gene pathway to be evaluated in experimental models of human diseases. The objective of this study was to investigate the feasibility and reproducibility of cross-species analysis employing a human cDNA microarray as probe.

**Results:**

As a proof of principle, total RNA derived from human and bovine fetal brains was used as a source of labelled targets for hybridisation onto a human cDNA microarray composed of 349 characterised genes. Each gene was spotted 20 times representing 6,980 data points thus enabling highly reproducible spot quantification. Employing high stringency hybridisation and washing conditions, followed by data analysis, revealed slight differences in the expression levels and reproducibility of the signals between the two species. We also assigned each of the genes into three expression level categories- i.e. high, medium and low. The correlation co-efficient of cross hybridisation between the orthologous genes was 0.94. Verification of the array data by semi-quantitative RT-PCR using common primer sequences enabled co-amplification of both human and bovine transcripts. Finally, we were able to assign gene names to previously uncharacterised bovine ESTs.

**Conclusions:**

Results of our study demonstrate the harnessing and utilisation power of comparative genomics and prove the feasibility of using human microarrays to facilitate the identification of co-expressed orthologous genes in common tissues derived from different species.

## Background

Microarrays are routinely used for large scale transcriptome analyses and have been widely and successfully employed for simultaneously monitoring the expression of a potentially unlimited number of genes in parallel, thus providing the basis for identifying genes differentially expressed in distinct cell-types, developmental stages, disease states and cells subjected to exogenous reagents [1]. The rapid and significant improvements of cDNA-chip technologies and the availability of multi-species gene catalogues within the various data bases have made possible the comparison of gene expression levels within a single mammalian organism and across different organisms on a large-scale.

The advantages of cross-species hybridisation are two-fold. First, cross-species gene-expression comparison is a powerful tool for the discovery of evolutionarily conserved mechanisms and pathways of expression control. The advantage of cDNA microarrays in this context is that broad areas of homology are compared and hybridization probes are sufficiently large so that small inter-species differences in nucleotide sequence would not affect the analytical results. This comparative genomics approach would allow a common set of genes within a specific developmental, metabolic, or disease-related gene pathway to be evaluated in experimental models of human diseases. Second, the use of microarrays in studies in mammalian species other than human and rodents, for example nonhuman primates, bovine, sheep and porcine may advance our understanding of human health and disease, for example the use of animal models in drug target validation. However, the inavailability of adequate sequence data and commercial cDNA and oligonucleotide microarrays keeps this technology beyond the reach of investigators working on economically and scientifically important large domestic species such as cattle, pigs and sheep. A potential solution to this problem is the use of cross-species hybridisations, i.e, human sequence-based arrays as tools for undertaking comparative genome expression studies. Such analyses have been performed using ape brain RNA as target on a human oligonucleotide array [2] and pig, mouse and Atlantic salmon RNA on human nylon arrays- [3-7]. These types of studies represent critical areas of research directly related to the understanding of human diseases because nonhuman primates, bovine, sheep and porcine play a crucial role in biomedicine, such as, organ transplantation, vaccine development, viral pathogenesis, gene therapy and a host of other human health-related technologies.

A crucial step employing domestic animals in biomedicine is genetic modification which requires extensive embryo and embryo-related technologies, such as *in vitro *production of embryos for stem cell derivation and somatic nuclear transfer cloning. Employing the bovine model and sensitive RT-PCR assays, it has been shown that the majority of embryos derived from such sources display distinct mRNA expression patterns in a variety of developmentally important genes compared to their *in vivo *derived counterparts [8]. Some of these aberrations lead to "Large offspring syndrome", a complex of multiple pathologies observed in offspring derived from *in vitro *production and/or nuclear transfer of which significant oversize is a predominant feature [9]. Analysis of mRNA expression patterns in early embryos via cDNA microarray technology would provide insights into the function of gene regulatory networks and would thus be a major step forward in unravelling molecular mechanisms underlying developmental abnormalities. The technology to amplify the minute amounts of mRNA in early embryos without significantly altering the ratio of the various mRNAs in the original cell has recently been described [10,11] and a prototype mouse cDNA-macroarray enriched in embryonic sequences has been developed [11].

Important criteria for evaluating any microarray system include the reproducibility of the data generated, the specificity of detection of the targeted gene, and the validity of the results that identify and establish differential gene expression. The experiments described here show the systematic validation of cross-species microarray analysis, with emphasis on the reproducibility and statistical analysis of generated data using standard microarray data analysis tools.

Specifically, we investigated the feasibility and reproducibility of cross-species hybridisation of orthologous genes within a defined developmental and metabolic pathway using as a test case and the first description of its kind, human and bovine fetal brain RNA as Cy-dye labelled targets on a human cDNA microarray. The microarray is composed of 349 genes each spotted 20 times to ensure reproducible validation by independent technologies such as semi-quantitative RT-PCR as carried out in this study, or alternatively, Real-Time PCR and Northern blot analysis.

## Results

### Array fabrication and gene annotation

**Figure 1:**
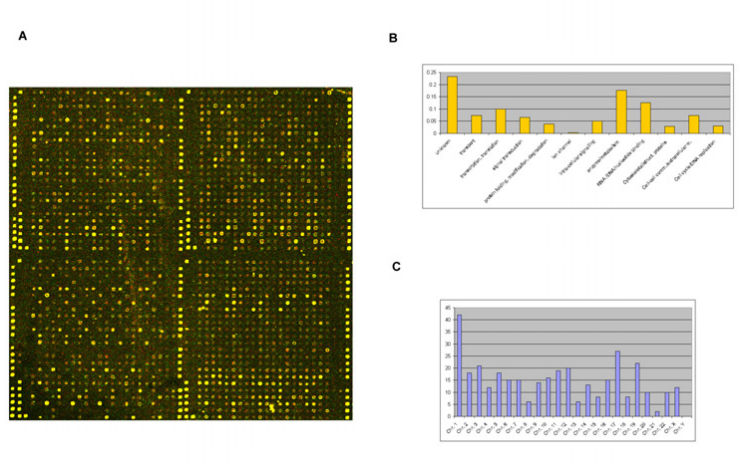
**Array fabrication and gene annotation **(A) False-colour image generated from a bovine-Cy5, human-Cy3 hybridisation. False colour images were generated using the programme ScanAlyze, version 2.44 . The full array (24 × 25 spotting pattern) consists of 16 blocks with each gene spotted 5 times per block therefore 80 potential data points present for expression analysis per gene. A blow-up of 4 blocks illustrating the Chip design is presented. The *ACTIN *cDNAs acting as guide-dots can be seen as intense yellow spots demarcating each block. In addition, the majority of spots appear yellow due to similar expression levels of the orthologous genes. (B) Functional annotation of the 349 genes as set out by the Gene Ontology Consortium .The proportion of these genes within each functional / biological annotation is represented on the Y-axis and the annotation on the X-axis. (C) Chromosomal distribution of the 349 genes. The number of genes within each chromosome is represented on the Y-axis and the chromosome numbers on the X-axis.

The human cDNA microarray used in this study consisted of 349 fully sequenced and annotated cDNAs as described in the Supplemental Table 1. Thirty-five spots containing only spotting solution (3x SSC / 1.5 M betaine) served as negative controls. In addition, in a separate control plate, the housekeeping genes *HPRT *and β-*ACTIN *(made up of a dilution series of 25-, 50-, 100-, 150- and 200 ng/μl) were employed as endogenous guide dots to enable accurate grid placement prior to image analysis. These spots can be seen as intense yellow signals at the periphery of each block as portrayed in Figure 1A. The embryonic-specific gene, *OCT-4 *and *Arabidopsis *cDNAs (*Cab*- T97312; *Cwlp*- T02614; *Lhb1B1*- T21965; *Ohp*- T22679) were included as negative controls to monitor hybridisation-specificity. The transcription factor *OCT*-4 is expressed in human embryonic stem cells and primordial germ cells and is down-regulated upon differentiation [10,12,13].

**Table 1:** Summary of primer sequences, annealing temperatures and size of amplicons. Derivation of primer sequences: H and B denote human and bovine respectively. The nucleotide positions of the primer sequences are in parenthesis. Nucleotides highlighted in bold denote differences in the orthologous gene sequences.

Genes	Primer sequences (5' – 3')	Annealing temperature (^°^C)	Fragment size (bp)	Accession numbers
CDC27	(1226–1250) ^H^ TATTACATCTCCCCCAAACGCACTG	54	311	NM_001256 ^H^ CB170694 ^B^
	(1512–1536) ^H^ CCATTTCACGAAGAAGGCTCATCAA			
CDC6	(89–113) ^B^ TCCCAAGCGGGTTGGT**G**TTATTCAC	54	208	NM_001254 ^H^ BF600055 ^B^
	(272–296) ^B^ GCGACAGACT**G**TACTGTAGGCTTCA			
PCNA	(195–217) ^B^ GAGGCGC**T**TAAGGATCTCA**T**CAA	54	382	AF527838 ^H^ CB531519 ^B^
	(554–576) ^B^ ATTCACCAGAAGGCATCTTTACT			
SLC11A3	(38–61) ^B^ ACCCCTGGAGGGAACTCATCTAAT	57	276	AF215636.1 ^H^ BG689712 ^B^
	(290–313) ^B^ GCTGATGCTCCCATCAAAATACTG			
VDAC2	(652–675) ^B^ CCT**C**GGTTGTGATGTTGACTTTGA	57	448	NM_003375.1 ^H^ TC152811 ^B^
	(1076–1099) ^B^ GTGGCCTCCAGCATTAATGCTCTT			
CALNEXIN	(1054–1078): ^H^ GCTGGTTAGATGATGAGCCTGAGTA	56	237	NM_001746.1 ^H^ BM431118 ^B^
	(1266–1290): ^H^ TCC**TG**GGTTTCCAGATTCCCTGGTA			
β-ACTIN	441–461) ^H^ GTTGCTATCCAGGCTGTGCT	60	469	NM_001101 ^H^ NM_173979 ^B^
	(890–910) ^H^ CGGATGTCCACGTCACACTT			

Classification of the genes according to Gene Ontology annotations (molecular function) and chromosomal location (Figures 1B and 1C) demonstrates that the selected genes encompass a range of twelve different functional classes and are located on all except the Y-chromosome. This implies that there is no obvious bias towards biological characteristics and the selected gene set can be viewed as representative for the current study.

### Global data characteristics

**Figure 2:**
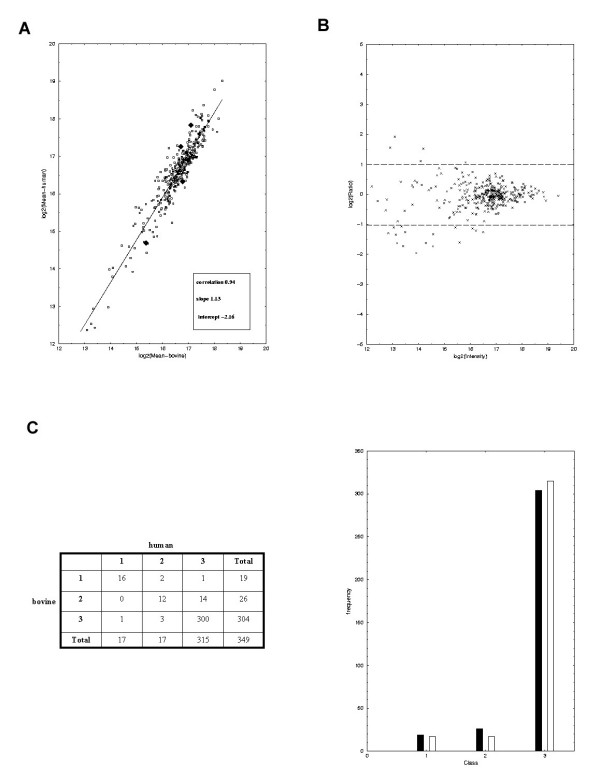
Global data characteristics (A) Global correlation of bovine (X-axis) and human (Y-axis) experiments. The plot shows a log-log (base 2) plot of the mean signal intensities from the four independent experiments for each gene. The red line shows the regression line, the box displays the parameters of the regression line (intercept, slope) and the overall correlation. Four genes that are significantly differentially expressed as judged by using the Wilcoxon's rank-sum test are denoted by black diamond shapes. (B) MA-plot of a single experiment. The X-axis show the log (base 2) of the squared product of Cy5-red and Cy3-green intensities of each gene in the same experiment. Y-axis shows the log-ratio (base 2) of red and green intensity. The horizontal lines mark the two-fold over-expressed genes (Top line; bovine over-expression and bottom line; human over-expression). (C). Classification of detection levels of the 349 genes. The genes were sub-divided into three classes of expression levels. Class 1 (BG-tag < 0.8) – low (not detected). Class 2 (BG-tag between 0.8 and 0.9) – medium (boarderline detection) and Class 3 (BG-tag > 0.9) – high expression (readily detectable) The number of genes (X-axis) within each class (Y-axis) is also shown in the figure (black = bovine, white = human).

Our experimental design incorporated dye-swaps and four replicated hybridisations. Three hundred and forty nine cDNAs, each representing a unique gene were spotted 20 times on each microarray. Four independent experiments were performed with human and bovine brain, respectively. Within each experiment the 20 replicates were averaged to yield a reliable signal for the respective probe. In the next step, the replicated signals from the different experiments were averaged to compute overall characteristics. The overall correlation of expression of the human and bovine genes is shown in Figure 2A. The regression line bears a slope close to one (1.13) thus indicating similar expression of human and bovine genes across the replicated experiments with a slight increase of expression in the human brain. The overall correlation coefficient is 0.94.

Additionally, we computed MA-plots [14] in order to detect artificial dependencies of the log-ratio across the signal range. Figure 2B illustrates a typical result from a specific hybridisation experiment. The horizontal lines mark the two-fold levels of over-expression in bovine and human, respectively. A clear observation is that a few of the 349 genes under investigation fall outside these thresholds with the expression of 5 genes being more than 2-fold over-expressed in bovine and 16 genes more than 2-fold over-expressed in human. However, there is no increase or decrease in fold-change for the vast majority of genes and this is fairly stable across the signal range.

In order to measure whether a given gene was significantly expressed, we compared its cDNA's signal to a signal distribution derived from negative controls represented by approx. 2,500 empty spot positions on the array. After quantification of each array a low non-zero intensity is assigned to each of these empty spots reflecting the amount of background signal on the array. Since these positions are spread uniformly over the array, the distribution of these signals reflects the distribution for signal noise and is an indicator whether signals are at the background level or reflect reliable expression levels. For each cDNA we counted the relative proportion of empty positions on the array that are smaller than the actual observed intensity (BG-tag). BG-tags from replicated experiments for the same cDNA were averaged. Thus, high values (close to one) indicate that the cDNA is expressed in the respective tissue whereas low values reflect noise. The limit of visual detection of a spot corresponds to a BG-tag level of 0.9, however there is a grey zone around this value. Comparison with RT-PCR analyses showed [15] that this level is consistent with the limit of detection at the 25^th ^cycle of a standard PCR reaction. cDNAs were considered as "detected" when their average BG-tag was above 0.9.

We grouped the 349 genes into three classes of expression levels with respect to their intensity values above background levels (i.e, BG-tag) with class 1 as low (not-detected), class 2 as medium (possibly detected) and class 3 as high level of expression (detected), respectively. Within class 1, are 19 bovine and 17 human genes. Class 2, comprises 26 bovine and 17 human genes and finally class 3 has 304 bovine and 315 human genes, respectively. The genes within expression class 1 have signal intensities below the detection level of our microarray analysis platform and as such these genes can be designated as "Absent" (See Figure 2C for a graphical illustration). A list of all the intensity values is given (see Additional file 1).

### Data reproducibility and species variability

**Figure 3:**
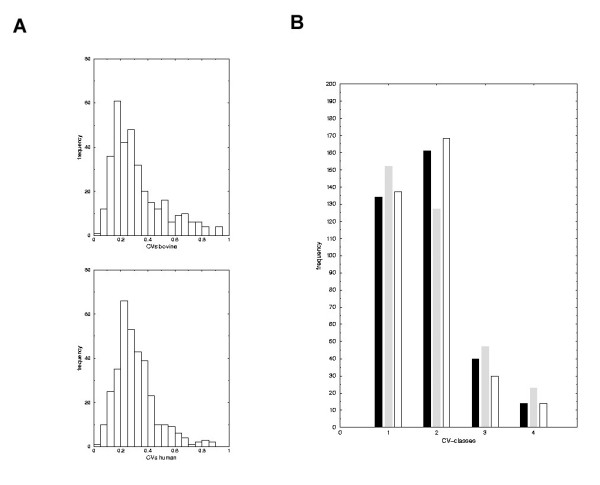
**Data reproducibility and species variability **(A) Histograms of CVs for human and bovine hybridisations. (B) Classification of signal variability in measured gene expression intensities. Human (white), bovine (grey) and mixed samples (black). CVs (co-efficient of variation) were calculated for repeated signal intensities of the four independent hybridisations and then sub-divided into four classes: Class 1 – CV < 0.25 high reproducibility, Class 2 – CV [0.25 – 0.5] good reproducibility, Class 3 – CV [0.5 – 0.75] medium reproducibility Class 4 – CV > 0.75 poor reproducibility.

An essential criterion for the applicability of cross-species experiments is the data reproducibility. For example, if human probes hybridise to bovine mRNA with far less reproducibility than to human mRNA high changes in expression of bovine tissues can be observed that are solely due to technical variability. In order to test whether the variability in gene expression levels is conserved within both species or in contrast, is higher in the bovine than in the human hybridisations, we calculated the coefficient of variation (CV) across the replicated experiments. Histograms are shown in Figure 3A. We then defined four classes of CVs for the genes (highly reproducible signals – CV < 0.25, good reproducibility – 0.25 < CV < 0.5, medium reproducibility – 0.5 < CV < 0.75 and poor reproducibility CV > 0.75). Note that a CV of 1.0 indicates that the signal standard deviation is in the order of the signal itself and therefore no meaningful statement on the measurement can be made.

Figure 3B depicts the number of genes that fall within the respective CV-classes when analysing all experiments (human and bovine- black), human only (white) and bovine only (grey). There is a 10% decrease in the number of reproducible genes when comparing human and bovine but the overall effect is similar. Approximately 305 genes show a CV<0.5 with human brain (87%) compared to 280 with bovine brain (80%). Only 15 genes (4%) show high variability across the experiments for human brain compared to 20 genes (5.7%) for bovine brain. Figure 3B illustrates that there is a slight but not dramatic increase in variability when performing cross-species hybridisation (the number of variable genes (class 3 and 4) increase by a factor of 1.5). However, for the vast majority of the genes the reproducibility is similar. The observed effect is not due to exact cutoff values set for the different CV-classes since a slight shifting of the class borders leads to the similar results. For example, shifting the borders about a slight factor of ϑ = ± 0.05 to the left/right respectively gives approximately the same factor of increase for variable bovine to variable human genes.

### Differential gene expression in bovine and human fetal brain

Three statistical tests that judge the significance of differences in the levels of gene expression in human and bovine fetal brains were employed (Student's t-test, Welch-test and, Wilcoxon's rank-sum test) as described previously [16]. Tests were carried out with the four independent experiments in bovine and human brain, respectively. Note that with independent experiments we mean the independent technical replicates since we do not employ different biological replicates in our study. Messenger RNA levels of seven genes were significantly (p < 0.05 with Student's t-test and Welch test) different between the two species, whilst four genes were found to be differentially expressed only with the Wilcoxon test (p < 0.05). This emphasizes that the Wilcoxon test is more conservative than the parametric tests. In contrast to the two parametric tests that assume a specific parametric signal distribution (Gaussian distribution) for the underlying signal series, the Wilcoxon test is non-parametric. Thus, P-values calculated by this test are valid in a more general set-up, i.e. for larger classes of probability distributions, than with the other tests. Furthermore, since the Wilcoxon test is based on ranks rather than on the underlying signals it is a more robust procedure in the sense that it is less sensitive against outliers from the model assumptions. These four genes are, *ZNF278*, *APOARGC*, *KIAA1609 *and *MGC12904 *– highlighted as diamond shapes in Figure 2A. The expression levels of the vast majority (98%) of the genes remained unchanged.

Furthermore, taking into account corrections for multiple testing, no gene is differentially expressed at the global experiment significance level of 0.05. For example, the lowest P-value of the Welch test is 1.98e-03, (*APOARGC*). Thus, Holm's setp-wise correction would start with an adjusted experiment level P-value of p = 0.05/349 = 1.43e-04. Similarly, measuring the false discovery rate by qvalues [17] results in no significant differential gene expression. Thus, we conclude that the level of expression of the individual 349 genes under investigation within human and bovine brain is roughly the same.

### Nucleotide sequence alignments of bovine and human transcripts

**Figure 4:**
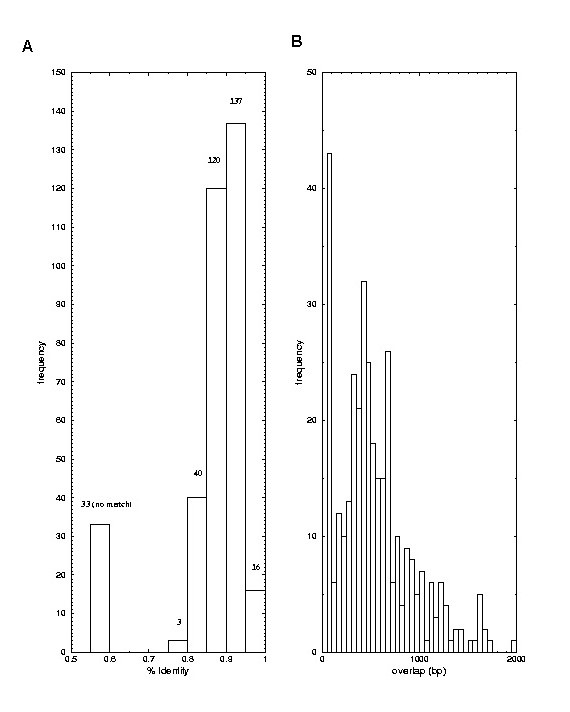
**Nucleotide sequence alignments of bovine and human transcripts **All 349 human genes were matched against the TIGR Bos taurus gene index, BtGI Release 8.0, which contains 87,257 unique sequences. (A) Histogram of %-identities with the best match. (B) Histogram of bp overlap with the best match.

In order to characterise some of the vast number of unknown bovine ESTs within the various databases we screened for orthologs to the human genes [18]. The 349 known human genes were screened against the TIGR Bos Taurus gene index (BtGI Release 8.0). Using high-quality matches (>85% identity, >100 bp overlap, E-value < 1.0e-15) we were able to ascertain the expression of 316 orthologous genes (see Additional file 1). Figure 4 shows the quality of the matches. Of these genes, 16 had sequence identities of greater than 95%, 137 genes with identities between 90% and 95%, 120 genes with identities of 85% to 90%. The remaining genes did not meet the criterion for the assignment as orthologs [18]. Forty genes had identities of 80% to 85%, 3 genes had identities between 75% and 80% and finally 33 genes did not have a significant BLAST hit. These matches are considered to be insignificant and therefore implying that these 33 human genes (assigned values (0) in Additional file 1) do not as yet have their bovine homologs present in the current bovine databases.

**Figure 5:**
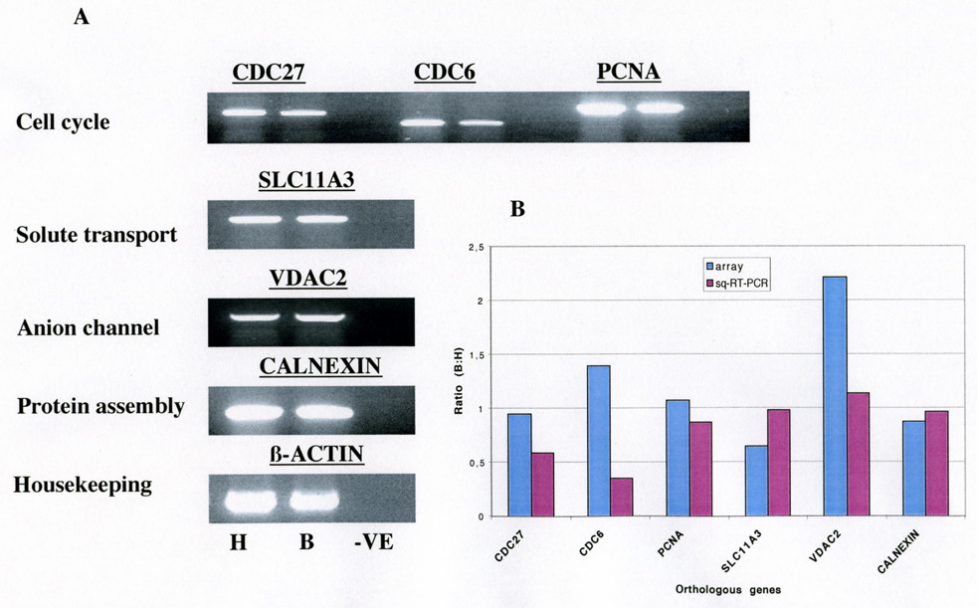
**Independent verification of array results by semi-quantitative RT-PCR **(A) Ethidium bromide stained gel illustrating independent verification of differential gene expression by semi-quantitative RT-PCR. PCR reactions were carried out in 50 μl volumes, loaded in the order H- (human), B- (bovine) and VE- (water as negative control) then resolved on a 2 % agarose gel as described in Materials and Methods. Each panel consist of genes of the same functional category – listed on the left hand side. A 100 bp ladder (Invitrogen) was used to confirm the sizes (Table 1) of the PCR products. (B) A graphical illustration of the comparison of the expression levels of the orthologous genes as deduced by the microarray analysis and semiquantitative RT-PCR.

Employing a comparative genomics approach we have functionally annotated previously uncharacterised bovine ESTs. These genes are depicted in the Supplemental Table as bovine ESTs lacking a gene name or description in the TIGR Bos Taurus gene index. As an example, the gene *SLC11A3 *which encodes a protein which functions as a solute carrier has 92% nucleotide sequence identity with an overlap of 500 bp with its bovine orthologue. Additionally, we have demonstrated co-amplification of this transcript in both human and bovine fetal brain RNA using primers derived from the bovine sequence – Figure 5A and Table 1.

### Verification of expression levels of cross-hybridised orthologous genes by semi-quantitative RT-PCR

Semi-quantitative RT-PCR was used to confirm the deduced expression data generated by the high stringency cross-species hybridisation. We selected a set of genes belonging to distinct families based on their published functional annotation, for example, cell cycle (*CDC27*, *CDC6 *and *PCNA*), solute transport- (*SLC11A3*), protein assembly- (*CALNEXIN*), anion channel- (*VDAC2*) and as an endogenous reference, the housekeeping gene, β-*ACTIN*. Primer sequences were designed to co-amplify the orthologous genes (Table 1).

The RT-PCR analyses (Figure 5A) using a common set of gene-specific primers clearly demonstrate co-amplifcation of the orthologous transcripts and, in addition, differences in expression levels between genes within the same species are discernable. For example, the house keeping gene β-*ACTIN *and *CALNEXIN *which is involved in protein assembly are more abundant than for examples genes involved in cell cycle control. Most importantly, the trends in the deduced ratios (see Additional file 1 and Figure 5B) of expression levels of orthologous genes from the hybridisation analysis are confirmed by the RT-PCR assay.

Discrepancies in the bovine-human ratio with array data and the semi-quantitative RT-PCR data are also observable. Furthermore, some of the genes (for example *CDC6 *and *VDAC2*) are expressed at a low level (expression class 1, compare Figure 2C). Here, microarray measurements are less reliable than PCR-based measurements and one comes close to the borderline of the detection limit of microarrays.

## Discussion

Customised and focussed microarrays containing orthologous genes related to function, tissue, or pathways are becoming widely adopted for studying mRNA expression patterns. In addition, the level of cross hybridisation between genes with high sequence identity is also of interest because arrays are not always available for mammalian species other than human and rodents so cross-species hybridisations are often carried out [19,20]. In view of this, it is crucial to know whether the hybridisation conditions (i.e. the stringency used for the hybridisation and subsequent washes) would enable identification of altered gene expression across species. Here we have demonstrated cross-hybridisation of orthologous transcripts by adopting a high stringency hybridisation and wash protocol.

The overall correlation co-efficient of gene expression in the fetal brains of human and bovine was 0.94, with the regression line at a slope of 1.13 (Figure 2A). This suggests that the 349 genes under investigation have rather similar expression levels as judged by the intensity values of the human and bovine genes across the replicated experiments. The use of replicate experiments is clearly essential in order to ascertain true expression change. For example, judging single experiments we find a couple of genes escaping the 2-fold bounds. The MA-plot [14] shown in Figure 2B suggests that of the 349 genes, 5 are over-expressed more than 2-fold in bovine and 16 are more than 2-fold over-expressed in human. However, most of the changes are due to experimental variability of this specific experiment.

An extremely important aspect of cross-species hybridisations is data reproducibility. A poor hybridisation experiment would lead to a high variability in the respective replicate experiments in human and bovine. With regards to this aspect, we identified fifteen genes (4%) in human and twenty genes (5.7%) in bovine with variability in expression levels in the four hybridisation experiments. In addition, approximately 305 genes have a CV of less than 0.5 with human brain (87%) compared to 280 with bovine brain (80%). However, this level of variability is low and can be expected due to the fact that the probe set used are of human origin. Moreover, the slight decrease in reproducibility due to species and nucleotide sequence differences can be compensated for by increasing the number of independent repetitions (biological and technical replicates) of the experiments. The unexpectedly low level of variability in gene expression levels between human and bovine fetal brain can also be attributed to the 80 data points examined per gene and the four replicate hybridisations (technical replicates) carried out for each species. Our finding further emphasises the need for sufficient technical and biological replicates in all microarray experiments.

In assessing differential gene expression in bovine and human fetal brain, we observed that the expression levels of the individual orthologous genes were roughly in the same broad range. For example, if one considers genes involved in pathways of cell cycle control, the microarray deduced ratios (bovine:human) were for *CDC27*: 0.95, *CDC6*: 1.36, *PCNA*: 1.07, respectively, whereas the deduced ratios derived from the semi-quantitative RT-PCR assay were 0.59 for *CDC27*, 0.35 for *CDC6 *and 0.87 for *PCNA*, respectively.

Similarly the comparative ratios for *SLC11A3 *and *CALNEXIN *are 0.65 vs. 0.98 and 0.88 vs. 0.97. An illustration of co-amplification with common primers and also the confirmation of the array data for a selection of genes can be seen in Figures 5A and 5B. The expression ratios of all the other genes under investigation are given (see Additional file 1). Though co-amplification is possible by semi-quantitative RT-PCR, there is amplification bias related to primer mismatch (See Table 1). This effect was further highlighted when primer efficiencies were calculated using comparative Real-Time PCR (data not included). An important difference between the two techniques is that cDNA array hybridisation is less sensitive to minor mismatches than PCR primer annealing. The bias in amplification specificity is not a drawback in this study and species specific primers would obviously be used to compare gene expression levels in an experiment in which a developmental/metabolic pathway or disease model is investigated.

The similar expression ratio of the orthologous genes implicated in cell cycle control and initiation of eukaryotic genome replication which is conserved in all eukaryotes highlights the feasibility and importance of our study in examining conserved pathways operative within the fetal brains of human and bovine and of course across other species using human cDNA microarrays. A similar finding has also been confirmed when comparing yeast co-regulated genes against the archaeal and bacterial operons. This implies that the components of the protein translation process are conserved across organisms at the expression level with minor specific differences [21].

We also identified four significantly differentially expressed genes as judged by the Wilcoxon test. The Wilcoxon test is known to be more conservative than the Student's t-test and the Welch-test, however, we have more confidence in this test since it is distribution-free, in particular it does not depend on the partly unrealistic assumption of an underlying Gaussian distribution.

The genes *ZNF278 *and *APOARGC *were 1.45 – and 1.66 -fold over-expressed in human whereas *KIAA1609 *and *MGC12904 *were 1.60 – and 1.37-fold over-expressed in bovine. *ZNF278 *encodes a zinc finger-containing transcription factor that acts as a transcriptional repressor and also implicated in small round cell tumours [22]. The gene *APOARGC *encodes a protein with hydrolase activity. *MGC12904 *and *KIAA1609 *are uncharacterised ESTs so comments cannot be made with regard to their function.

Orthologous genes between human and mouse and between human and rat both have a mean of approximately 85% sequence identity [18,23]. In two independent and unrelated studies carried out on cDNA and 50-mer oligonucleotide microarrays, cross-hybridisation was only observed with genes with 70%-80% and 50%-75% overall sequence identity, respectively [24,25]. With respect to these studies, our data unequivocally confirm the feasibility and reproducibility of cross-species hybridisation of orthologous genes within defined developmental and metabolic pathway(s) operative in human and bovine fetal brains. In addition, we have been able to assign gene names to previously uncharacterised bovine ESTs, thus, highlighting the importance of comparative genomics in identifying orthologous genes across species.

Furthermore, using this protocol of cross-species hybridisation, we have compared gene expression in bovine unfertilised oocytes and blastocyst using a larger microarray (The Human Ensembl Chip) comprising 15,500 fully sequenced and annotated genes and ESTs. The correlation co-efficient between the RNA samples is 0.26, thus reflecting the diversity between oocyte derived maternal transcripts and embryonic transcripts derived from the blastocyst (Adjaye et al., unpublished).

## Conclusions

In summary, this study highlights the significance and utilisation power of comparative genomics and also demonstrates the feasibility of using human cDNA microarrays to facilitate the identification of differentially expressed genes in human and bovine fetal brain. Our results indicate that cross-species hybridisation is not only a useful short-term solution for studying species for which gene maps, cDNA or oligo microarrays are not yet available, but also possesses tremendous power in enabling the unravelling of common evolutionary evolved mechanisms in different species.

## Methods

### Microarray fabrication

For the generation of probes for spotting, cDNA inserts from a single 384-well plate of non-redundant, fully sequenced, annotated human cDNA collection (Human Ensembl set RZPD1.1) were employed- (BenKahla et al., unpublished) and a second partial plate consisting of control genes and empty wells to be used for data normalisation were amplified by PCR in a 384-well format. Bacterial clones were transferred to the PCR plates using 384-well replicators (Genetix Ltd, New Hampshire, UK). PCR amplifications were carried out in a total volume of 25 μl consisting of 1x PCR buffer, 1.5 M Betaine (Sigma, Germany), 1U Taq polymerase, 200 mM of each dNTP (Invitek, Germany), 25 pmoles of M13-forward (5'GTAAAACGACGGCCA3') and M13-reverse (5'CAGGAAACAGCTATGAC3') primers respectively. Cycling parameters typically consisted of an initial denaturation step at 95°C for 5 min followed by 35 cycles of denaturation at 95°C for 1 min, annealing at 55°C for 1 min, elongation at 72°C for 1 min, then a final elongation step at 72°C for 10 min. All amplified products were analysed by agarose gel electrophoresis and in all cases single bands were obtained per gene.

Purification was carried out using isopropanol precipitations. In brief, 24 μl of the PCR product was transferred into 384-well Genetix plates (Genetix Ltd, New Hampshire, UK) previously filled with 18 μl of isopropanol per well. The samples were mixed by gentle vortexing after sealing the plates with transparent tape. After precipitation overnight at -20°C, the plates were spun at 3,500 rpm for 2 hrs at 21°C, the isopropanol decanted off and the plates dried briefly in a SpeedVac without heating. The pellets were resuspended in 6 μl of spotting solution (3x SSC /1.5 M Betaine (N, N, N-trimethylglycine; Sigma, Germany)). Random sampling of cDNAs revealed concentrations ranging between 200 to 300 ng/μl. Finally the samples in each well were spotted twenty times (for signal averaging) with four slit pins arranged in a 2x 2y printhead (Chipmaker 2 pins, Telechem, Sunnyvale, CA, USA). Prior to every spotting run the performance of all components of the robot is carefully tested – in particular the performance of the washing station in test runs designed to detect possible sample carry over. A modified Genetix Q-array (Genetix Ltd, New Hampshire, UK), controlled by a novel in-house software was used for the arraying of the samples on SuperAmine™ aminosilane-coated microscope slides (Telechem, Sunnyvale, Ca, USA). Post-processing of the arrays was performed using 1,2-dichloroethane and the acylating catalyst N-methylimidazole following protocols described previously [26].

### Isolation of total RNA

Human brain RNA was isolated from a medically terminated fetus at 10 weeks gestation. This was provided by the MRC-funded Human Embryonic Tissue Bank maintained at the Institute of Child Health and University College London, England. Bovine fetal brain was obtained from a 3–4 months old bovine fetus at a local abattoir near Mariensee, Germany. Immediately after slaughter of the pregnant female, the tissue was plunged into liquid nitrogen and transported to the laboratory. Brain tissues were homogenised using a Dounce homogeniser, total RNA isolated using TRIzol reagent (Invitrogen) and further purified using phenol/chloroform extractions and precipitation with ethanol after DNase 1 (Promega) treatment. All procedures were as described by the manufacturers. RNA Purity, integrity and concentrations were evaluated on the Agilent 2100 Bioanalyzer. High quality RNAs with A260/A280 ratio over 1.8 with intact ribosomal 28S and 18S RNA bands were utilised for subsequent labelling reactions.

### Direct labelling of RNA and hybridisations

Four independent labelling (including dye-swaps) reactions per species were carried out using 25 μg total RNA from both human and bovine per labelling reaction. Direct incorporation of Cy3 and Cy5 during reverse transcription was carried out in a 20 μl reaction volume using 1 μg of anchored oligo-dT primer. The RNA/primer mix was incubated at 70^°^C for 5 min., left at room temperature for 10 min and then cooled on ice for 2 min. The following reagents were then added: 4 μl first-strand buffer, 2 μl 0.1 M DTT, 0.5 μl dNTP mix (25 mM each of dATP, dGTP, dCTP and 10 mM dTTP), 1 μl of 1.0 mM Cy5- or Cy3-dUTP (Amersham Pharmacia) and 1 μl (200 U/μl) Superscript II (Invitrogen). The labelling reaction was carried out at 42^°^C for 1.5 hrs. The reaction was stopped with 4 μl of 0.5 M EDTA. The input RNA was hydrolysed by the addition of 2 μl of 2.5 M NaOH and incubated at 37^°^C for 15 mins. followed by neutralization with 10 μl HEPES free acid (2 M, pH 5.5). Labelled cDNAs (four replicates per species which included dye-swaps) were purified from unincorporated Cy-dyes using Microcon YM-30 purification columns (Millipore). All labelled cDNAs were routinely analysed on a Fuji scanner (FL8-8000) and by ethidium bromide staining to ascertain dye incorporation and size-range of synthesized cDNAs. After concentrating cDNAs by evaporation in a SpeedVac, labelled targets were resuspended in 20 μl of hybridisation buffer (10 μg polydA and 20 μg Human Cot1 DNA,-Invitrogen; DIG-Easy Hybridisation mix -Roche). After thorough resuspension, the cDNA was denatured by heating at 95^°^C for 5 mins followed by 20 mins. at 42^°^C to enable annealing of the blocking reagents to repetitive sequences within the target cDNAs. The hybridisation mixture consisting of Cy3-labelled bovine and Cy5-labelled human RNA and vice versa was placed on the blocked array under a 24 × 40 mm coverslip (Menzel-Glaser, Germany). To maintain humidity inside the chamber, 20 μl of 3x SSC was added to the reservoir wells. The chamber was then tightly sealed and slides incubated at 42^°^C for 18 hrs in a waterbath. Slides were washed twice in 0.2x SSC / 0.1% SDS and then twice in 0.2x SSC. Washes were carried out at room temperature with 10 min durations per wash. Finally, the slides were dried by centrifugation at 1100 rpm for 10 min.

### Image acquisition and data analysis

Fluorescence images were captured using an Affymetrix 428 scanner (Affymetrix, Santa Clara, CA) with appropriate gains on the photomultiplier tube (PMT) to obtain the highest intensity without saturation. A 16 bit TIFF image was generated for each channel for subsequent image analyses. Image analysis was carried out by placing the centre of each spot manually (grid-finding step) using the software AIDA (Raytest-Germany) and then by quantifying in a pre-defined neighbourhood around this spot centre using a two-dimensional Gaussian distribution (quantification step).

Data analysis comprised two distinct parts. In the first part, data were normalised to eliminate extrinsic influencing factors and artefacts not attributable to the probe-target interaction. For each tissue (human and bovine brain) under analysis the whole batch of experimental replicates was normalized simultaneously. Normalisation should eliminate multiplicative technical bias between the different experiments and result in the same median signal level for each experiment. In a first step, the local background of each spot was subtracted from the spot's signal intensity. Then, for each experiment *j *the median signal, med_j_, was computed and a multiplicative factor was calculated according to med_ref_/med_j_, where med_ref _is the global derived from the average intensity values of the cDNAs across the full batch of experiments. The multiplicative factor was used to adjust the signal of the *i*th cDNA in the *j*th experiment, x_ij_, by x_ij _* med_ref_/med_j_.

In the second part, a number of numerical characteristics were calculated in order to quantify the cross-species comparison. These characteristics were signal detection value, signal reproducibility and statistical significance of differential expression. Signal detection was judged by a number of empty positions that were spread across the array. For each experiment, the proportion of signals of empty positions lower than the actual spot signal was calculated. Then across all repetitions, the average proportion was kept as the signal detection value. Employing this procedure, a signal strength was quantified for each cDNA. Signal reproducibility was judged by calculating the co-efficient of variation (CV) for each cDNA across all experiments.

In order to judge differential expression of a gene in human and bovine brain we calculated three statistical tests: Student's t-test, Welch-test and Wilcoxon's rank sum test for each cDNA based on the signal series derived from human and bovine experimental repetitions. The three tests make different assumptions on the signal series. Whereas the first two assume that the series are Gaussian distributed, the Wilcoxon test is distribution-free. Low P-values calculated by these tests indicate significant differences in signal intensity within the human and bovine samples [15].

### Semi-quantitative RT-PCR analysis

The list of primers, annealing temperatures and genes under investigation is shown in Table 1.

For primer annealing and reverse transcription, 1.0 μl (2.5μg/μl) of DNase1 treated human and bovine total RNA was added to 2.0 μl (50 μM) Oligo-dT primer plus 9.0 μl of RNAse free water. The mixture was spun briefly and heated to 70^°^C for 5 mins and cooled on ice. Thereafter, the following components were added sequentially, 4.0 μl of 5x RT. Buffer (Invitrogen), 2.0 μl of 0.1 M DTT (Invitrogen) 1.0 μl of (10 mM) dNTP (Amersham) and 1.0 μl (200 U/μl) Superscript II (Invitrogen). After pulse spinning, incubation was carried out at 42^°^C for 1.5 hrs.

Gene-specific PCR amplifications were carried out in a total volume of 50 μl consisting of 5 μl of 10x PCR buffer, 0.2 μl (5 U/μl) Taq Polymerase, 1.0 μl (10 mM) dNTP, 2.5 μl of each (20 μM) primer, 1.5 μl (50 mM) MgCl_2_, 2.0 μl of the first strand cDNA (equivalent to 125 ng of input RNA) and distilled water to 50 μl. All reagents were purchased from Invitrogen. Cycling parameters consisted of an initial denaturation step at 95°C for 5 min followed by 30 cycles of denaturation at 95°C for 30 sec, annealing for 30 sec, elongation at 72°C for 30 sec, then a final elongation step at 72°C for 5 min. Primer sequences, annealing temperatures and predicted size of cDNA products are shown in Table 1. The amplification reaction was carried out in a PTC 200 PCR machine (MJ Research). After PCR amplification, 50 μl of the reaction products were resolved on 2.0 percent agarose gel containing 0.2 μg/ml ethidium bromide. The gel was placed on a U.V. transilluminator (UVP Model TFM-20, Ultra-Violet Products Ltd., Cambridge, U.K.) with 25-watt UV tubes for high fluorescence and high sensitivity on stained gels), and imaged with a Photometrics Quantix 1401E, 12-bit cooled CCD camera (Roper Scientific GmbH, Ottobrunn, Germany). Densitometric analysis of the digitized image was performed with the IPLab-Gel program from Scanalytics (Fairfax, VA, U.S.A.). The semi-quantitative RT-PCR assay provides sensitive and reliable results, (26). The linear range of amplification was determined by product quantification after different cycle numbers. Identical amounts of bovine and human mRNA were amplified with the same number of PCR cycles and were always placed next to each other on the gel. Each RT-PCR was repeated at least three times for each gene. The housekeeping gene β-*ACTIN *was used as endogenous control.

## Authors' contribution

J.A. conceived the study's idea, designed and optimised the protocols, carried out the hybridisations and was pivotal in developing the analysis plan and writing the manuscript.

R.H. developed the analysis plan, analysed the data and was pivotal in writing the manuscript.

D.H and M.N designed the cross-species primers and carried out the semi-quantitative RT-PCR.

W.W. performed the initial image analysis.

A.BK. selected the clones used as probes for creating the Chip and carried out the assignment of orthologous genes.

T.B. assisted in the semi-quantitative RT-PCR analysis

J.W.C. was involved in the conceptualisation and writing

C.H. was instrumental in the Chip design and production.

H.N. is the Head of the Department of Biotechnology at the Institute for Animal Science and was involved in the conceptualisation and writing.

H.L. is the Head of the Department of Vertebrate Genomics at the Max Planck Institute for Molecular Genetics.

## Supplementary Material

Additional File 1 **Summary of the expression data and the corresponding gene annotations**. The terms used under each column are explained viz;
IMAGE ID: Gene specific identifier assigned by the IMAGE consortium
Gene: HUGO gene name
Chromosome: Chromosomal location of gene
Description: Gene description
Molecular Function: Assigned function by the Gene Ontology Consortium
BG-tag-bovine: Signal detection probability in the bovine samples
BG-tag-human: Signal detection probability in the human samples
CV-bovine: Co-efficient of variation between replicate signal intensities
CV-human: Co-efficient of variation between replicate signal intensities
Mean-bovine: Mean signal across all hybridisation experiments using bovine RNA
log-Mean-bovine: Logarithm (base 2) of the mean value
Mean-human: Mean signal across all hybridisation experiments using human RNA
log-Mean-human: Logarithm (base 2) of the mean value
Ratio(bovine vs human): Ratio of the mean values
log2(Ratio): Log-ratio (base 2) of the mean values
P-value-Student's-t-test: P-value of Student's t-test using the replicate experiments
P-value-Welch-test: P-value of the Welch test using the replicate experiments
P-value-Wilcoxon-test: P-value of Wilcoxon's test using the replicate experiments
Match: A BLAST hit with E-value < 1.0e-15 was found (1) or not (0)
Homology: Classification of sequence homology between human gene sequence and bovine ESTs. "ortholog" = best match in both directions, "paralog" = bovine EST has its best match with another human sequence.
TIGR-BTGI0-51503- Matches: Best results of the BLAST matches using the Ensembl- annotated gene sequence with the TIGR- database
Identity: %-identity of best match
Overlap: Overlap in base pairs Click here for file
